# *In vitro* and *in vivo* assessment of CYP2C9-mediated herb–herb interaction of Euphorbiae Pekinensis Radix and Glycyrrhizae Radix

**DOI:** 10.3389/fphar.2014.00186

**Published:** 2014-08-22

**Authors:** Xinmin Wang, Yunru Peng, Xinyue Jing, Dawei Qian, Yuping Tang, Jin-ao Duan

**Affiliations:** ^1^Jiangsu Collaborative Innovation Center of Chinese Medicinal Resources Industrialization and Jiangsu Key Laboratory for High Technology Research of TCM Formulae, Nanjing University of Chinese MedicineNanjing, China; ^2^National and Local Collaborative Engineering Center of Chinese Medicinal Resources Industrialization and Formulae Innovative Medicine, Nanjing University of Chinese MedicineNanjing, China; ^3^Jiangsu Provincial Academy of Chinese MedicineNanjing, China

**Keywords:** Glycyrrhizae Radix, Euphorbiae Pekinensis Radix, CYP2C9, herb–herb interaction, traditional Chinese medicine

## Abstract

According to traditional Chinese medicine theories, Euphorbiae Pekinensis Radix and Glycyrrhizae Radix should not be used together in one prescription, because their interaction leads to an unexpected consequence. However, the mechanism remains unclear. The purpose of this study was to find out whether CYP2C9 was involved in this herb–herb interaction by using tolbutamide as a probe substrate *in vivo* and *in vitro*. Both Euphorbiae Pekinensis Radix and Glycyrrhizae Radix showed induction activity toward CYP2C9, while the combination of them showed a more potent induction activity toward CYP2C9 *in vivo*. *In vitro* study revealed only the combination of the herbs could induce the activity of CYP2C9. Thus, both *in vivo* and *in vitro* study indicated combination of Glycyrrhizae Radix and Euphorbiae Pekinensis Radix could induce the activity of CYP2C9 to a high level, which may result in decreased plasma levels of major active ingredients of these two herbs, as well as other herbs in the prescriptions. Further research also appears to be necessary to identify the main enzymes involved in the metabolism of the active ingredients in Glycyrrhizae Radix and Euphorbiae Pekinensis Radix.

## INTRODUCTION

Euphorbiae Pekinensis Radix is the dried roots of *Euphorbia pekinensis* (EP) Rupr., which belongs to the Euphorbiaceae family (Tao et al., 2013a). Pharmacological investigations demonstrated that its extract exhibited a variety of biological effects, including possess cytotoxic, antivirus, and anti-inflammatory activity (Mucsi et al., 2001; Corea et al., 2004; Hou et al., 2013). However, it is considered as a toxic herb in Chinese medicine. Glycyrrhizae Radix, the root of *Glycyrrhiza uralensis* (GU) Fisch., or *G. glabra* L., or *G. inflata* Bat., Leguminosae, is one of the oldest and most popular herbal medicines in the world, and it is recorded in the pharmacopeias of many Asian and European countries, including China, Japan, and the UK (Tao et al., 2013b). Extensively used in traditional Chinese medicine (TCM), it appears as a component herb in approximately 60% of all TCM prescriptions (Zhang and Ye, 2009) because most TCM practitioners in ancient and modern times commonly believe that Glycyrrhizae Radix may harmonize and modify other herbs (Shen et al., 2013). However, according to TCM theories, Euphorbiae Pekinensis and Glycyrrhizae Radix are prohibited to be used together in TCM clinics, which has been recorded in each edition of Chinese Pharmacopeia (Commission, 2010). From the previous reports, the side effects of this herb–herb interaction might be classified into three types including acute and predictable reactions, idiosyncratic reactions and cumulative, chronic, or delayed toxicity (Lee et al., 2013). Until now, the mechanism of this herb–herb interaction remains unclear.

A number of studies have reported many herbal interactions due to cytochrome P-450 enzymes (CYP450) which can mediate drug elimination as a major mechanism responsible for such types of interactions. CYP450 constitute a large family of cysteinato-heme enzymes, and are present in all forms of life (plants, bacteria, and mammals; Meunier et al., 2004). These enzymes are potent oxidants that are able to catalyze the hydroxylation of saturated carbon hydrogen bonds, the epoxidation of double bonds, the oxidation of heteroatoms, dealkylation reactions, oxidations of aromatics, and so on (Meunier et al., 2004), thus play a key role in the oxidative transformation of numerous endogenous compounds as well as xenobiotics including drugs (de Montellano and De Voss, 2002). They are well known to be composed of more than 400 isoforms. Among them, CYP2C9 has been considered as one of the most important drug-metabolizing enzymes subtypes in human, which is vital for the hydroxylation of 10–20% of commonly prescribed drugs (Al-Jenoobi, 2010; Yu et al., 2014). According to previous reports, CYP2C9 catalytic activities can be inducted or inhibited by many drugs, as well as TCM, thus giving rise to drug–drug interactions (Schmidt et al., 2007) and drug–herb interactions (Kennedy and Seely, 2010).

In the previous reports, there is a lack of information about the potential interactions of herbs, not to mention the limited availability of herb–herb interaction studies involving CYP2C9. The aim of this study was to investigate the potential mechanism of the incompatibility of Euphorbiae Pekinensis and Glycyrrhizae Radix from the field of drug-metabolizing enzymes CYP2C9.

## MATERIALS AND METHODS

### CHEMICALS AND REAGENTS

Tolbutamide, 4-hydroxytolbutamide, chlorpropamide, glucose 6-phosphate (G 6-P), glucose 6-phosphate dehydrogenase (G 6-PDH, Type V), and nicotinamide adenine dinucleotide phosphate (NADP) were purchased from Sigma Chemical Co. (St Louis, MO, USA). Acetonitrile (HPLC grade) was purchased from TEDIA Company Inc. (Fairfield, USA); formic acid was obtained from Merck KGaA (Darmstadt, Germany); ultra-pure water was purified by an EPED super purification system (Nanjing, China). The distilled water was used for the extraction and preparation of samples. Other reagents and chemicals were of analytical grade.

### MATERIALS AND EXTRACT PREPARATION

The dried roots of EP were collected in May 2010 from Nanning City, Guangxi province, China, and the dried roots and rhizomes of GU were collected in December 2010 from Lingwu City, Ningxia Province, China. The two herbs were identified by Professor Chungen Wang (Department of Pharmacognosy, College of Pharmacy, Nanjing University of Chinese Medicine, Nanjing, China).

The dry herb pieces of EP (500 g) and GU (500 g) were mixed, and then extracted with boiling water (1:10, w:v) for twice, 2 h for each time, filtered through gauze. The two filtrates were merged and evaporated by rotary evaporation under vacuum at 60°C, thus the water extract of EP (WEP)-GU (WEP-GU) were obtained. Its concentration was set at 2 g/mL (2 g crude herbs per 1 mL) using pure water. The single dry herb pieces of EP (500 g) and GU (500 g) were extracted through the same procedure, and then 1 g/mL WEP and GU (WGU) were obtained, respectively. Additionally, the dry herb pieces of EP (500 g) were refluxed with 90% ethanol under the same extract conditions as WEP-GU. The resulting extract was evaporated to dryness under reduced pressure, and was diluted in distilled H_2_O. Finally, 1 g/mL alcohol extract of EP (AEP) were prepared. All prepared solutions were stored at 4°C.

### ANIMALS AND HERBS ADMINISTRATION

Seventy two normal male Sprague–Dawley rats (SPF) weighing 200–250 g were purchased from Shanghai Slac Laboratory Animal Co. Ltd. (Shanghai, China). The rats were housed in a room with controlled conditions (temperature 20–25°C, relative humidity 55–60% and 12 h dark–light cycle), and allowed to freely access to food and water for 7 days acclimation. Animal study was carried out in accordance with the Guideline for Animal Experimentation of Nanjing University of Chinese Medicine, and the protocol was approved by the Animal Ethics Committee of the institution. All efforts were made to ameliorate suffering of animals.

The rats were randomly divided into six groups with 12 rats in each: Control, WEP, WGU, WEP-GU, AEP, and AEP-WGU groups. The rats in WEP, WGU, and AEP groups were intragastrically given WEP, WGU, and AEP extracts at a dose of 10 g/kg (10 g crude herbs per 1 kg rat body weight). WEP-GU group were oral administration of WEP-GU at a dosage of 20 g/kg, and AEP-WGU group were given the mixed solutions of WGU (10 g/kg) and AEP (10 g/kg). The animal dose of WEP, WGU, WEP-GU, AEP, and AEP-WGU extracts was extrapolated from the human daily dose, according to human dosage in clinical practice and human–rat coefficient of skin surface area. The dose of WGU, WEP extracts were equivalent to ten and fifty times of the adult daily dose crude herbs based on the TCM prescription, respectively. Control group was intragastrically given the same volume of saline solution. All animals were administered by oral gavage one time each day for continuous 10 days and fasted for 12 h before the experiments.

### BLOOD SAMPLING

For 6 rats of each group, tolbutamide was injected via caudal vein at a dose of 10 mg/kg 24 h after the last treatment of herbal extracts and blood samples (about 400 μL) were immediately collected in heparinized 1.5 mL polythene tubes from the suborbital vein at 0.167, 0.333, 0.667, 1.5, 4.0, 3.5, 4.0, 6.0, 8.0, and 22 h after injection. Within 30 min after blood withdrawal, the samples were centrifuged at 5000 rpm for 10 min and the separated plasma samples were stored at -80°C prior to analysis.

### PREPARATION OF PLASMA SAMPLES

A simple liquid–liquid extraction method was followed for extraction of tolbutamide and 4-hydroxytolbutamide. A 200 μL volume of plasma sample was transferred to a 1.5 mL PE tube, 50 μL internal standard solution (chlorpropamide dissolved in methanol at a concentration of 30 μg/mL) was spiked and vortexed for 1 min, followed by adding 1.0 mL absolute ether. After vortexing for 1.0 min, samples were centrifuged at 13,000 rpm for 10 min. The super layer was transferred to a 1.5 mL PE tube and evaporated to dryness at 30°C under a slight stream of nitrogen. The residue was reconstituted in 120 μL of methanol and vortex mixed for 3 min, followed by centrifugation at 13,000 rpm for 10 min. Finally, a 10 μL aliquot of the supernatant was injected for HPLC–UV analysis.

### MICROSOMES PREPARATION

The other six rats of each group were sacrificed 12 h after the last treatment and the livers were immediately removed, weighed, and washed in cold phosphate buffer saline (50 mM Tris–HCl, 150 mM KCl, 2 mM EDTA, pH 7.4). The liver was then homogenized in 3 mL buffer per gram of liver. The homogenates were submitted to several differential centrifugations, as previously described (Richert et al., 2002). Microsomal samples were finally aliquoted and frozen at -80°C until analysis. Protein content was determined by the method of Bradford (Bradford, 1976), and BSA was used as a standard.

### MICROSOMAL INCUBATION

The incubation mixture (final volume: 200 μL) consisted of an NADPH-generating system (0.5 mM NADP, 5.0 mM G-6-P, 1.0 U/mL G-6-P-OH, 5.0 mM MgCl_2_), 0.1 M phosphate buffer (pH 7.4), 0.5 mg/mL rat liver microsomes and 100 μM tolbutamide. After a 5 min pre-incubation at 37°C, the reaction was initiated by adding NADP. The reaction was terminated by adding 200 μL cold acetonitrile (containing internal standard chlorpropamide, 100 ng/mL) and placing the tubes on ice after incubation at 37°C for 50 min. Preliminary experiments showed that the formation of 4-hydroxytolbutamide was linear against incubation time for up to 60 min for 0.5 mg/mL of rat hepatic microsomes.

### PREPARATION OF MICROSOMAL SAMPLES

Microsomal samples were centrifuged at 13,000 rpm for 10 min and a 10 μL aliquot of the supernatant was injected for UPLC–MS/MS analysis.

### ANALYSIS OF BLOOD SAMPLE

Analysis of blood sample was performed using a Waters 2695 Alliance HPLC system (Waters Corporation, Milford, MA, USA), consisting of a quaternary pump solvent management system, an on-line degasser, and an autosampler. The raw data were detected by 2998 PDA, acquired, and processed with Empower^TM^ Software. An Agilent Extend-C18 column (4.6 mm × 150 mm, 5 μm) was applied and detection wavelength was set at 220 nm for all analyses. The mobile phase was composed of A (water) and B (acetonitrile) with a linear gradient elution: 0–8 min, 15–35% B; 8–10 min, 35% B; 10–10.5 min, 35–15% B; and 10.5–13.5 min, 15% B. The flow rate of the mobile phase was 1.0 mL/min, and the column temperature was maintained at 30°C.

### ANALYSIS OF MICROSOMAL SAMPLE

Analysis of microsomal sample was performed using a Waters ACQUITY UPLC system (Waters, Milford, MA, USA). An Acquity BEH C18 column (2.1 mm × 100 mm, 1.7 μm) maintained at 30°C was used with an injection volume of 2 μL. Mobile phase A was a 0.1% formic acid/water solution (1/1000, v/v), mobile phase B was a acetonitrile solution, and the flow rate was 0.4 mL/min. The linear gradient conditions were: 0–2 min, 30–90% B; 2–2.5 min, 90% B; 2.5–3.0 min, 90–30% B; and 3.0–3.5 min, 30% B.

Mass spectrometric analysis was carried out using a Waters Xevo TQ tandem quadrupole mass spectrometer (Micromass MS Technologies, Manchester, UK). All of the target compounds were detected using an ESI ion source. The parameters in the source were set as follows: capillary voltage = 3.0 kV; source temperature = 150°C; desolvation temperature = 550°C; cone gas flow = 50 L/h, and desolvation gas flow = 1000 L/h. Quantification was performed using multiple reaction monitoring (MRM) of the transitions *m/z* 270.58 → 73.97, *m/z* 287.16 → 73.97, and *m/z* 275.03 → 125.86 for tolbutamide, 4-hydroxytolbutamide, and IS, respectively. All data were acquired and processed using the Masslynx 4.1 software.

The method was validated in terms of specificity, lower limit of quantification (LLOQ), linearity, accuracy, precision, recovery, matrix effect, and stability, in according with the currently accepted USA Food and Drug Administration (FDA) bioanalytical method validation guidelines and the subsequent 2006 Bio-analytical Methods Validation Workshop white paper.

### STATISTICAL ANALYSIS

The corresponding pharmacokinetic parameters were calculated by the software of DAS 2.0 (Pharmacology Institute of China). Data were expressed as mean ± SD. ANOVA was used as statistical methods to evaluate the effects of the different treatment.

## RESULTS

### VALIDATION OF THE ANALYTICAL APPROACH

Under the conditions described in the experimental section, the validated HPLC–UV method and UPLC–MS/MS method were used to determine the levels of the probe substrate (tolbutamide for CYP2C9) and its metabolites in rat plasma and in the incubation system after different treatment for 10 days, respectively.

Standard curves of tolbutamide and 4-hydroxytolbutamide in rat plasma were established over the concentration range of 97.65–50,000 ng/mL and 97.65–6250 ng/mL, respectively. The calibration curves were: *y* = 0.097*x* + 0.021 (*r*^2^ = 0.9991, *n* = 5) for tolbutamide and *y* = 0.072*x* + 0.001 (*r*^2^ = 0.999, *n* = 5) for 4-hydroxytolbutamide (*y* = concentration of the analyte; *x* = peak area ratio of each analyte versus internal standard).

Standard curves of 4-hydroxytolbutamide in the incubation system was established over the concentration range of 1–10,000 ng/mL and the calibration curves were: *Y* = 4.147*X* + 0.150 (*r*^2^ = 0.999, *n* = 5; *Y* = concentration of 4-hydroxytolbutamide; *X* = peak area ratio of 4-hydroxytolbutamide versus internal standard).

The results of linear regression analysis showed that the correlation coefficients of the calibration curves for all sample types were above 0.996. The limit of quantifications of tolbutamide and 4-hydroxytolbutamide in rat plasma were both 97.65 ng/mL, and that of 4-hydroxytolbutamide in incubation system were 1 ng/mL. The precision and accuracy of intra-day and inter-day of all the analyte for the low-, medium-, and high-quality control samples were below 15%. The result of the chromatographic validation showed that the assay methods were suitable for this study.

### RESULT OF *IN VIVO* EXPERIMENT

**FIGURE 1:**
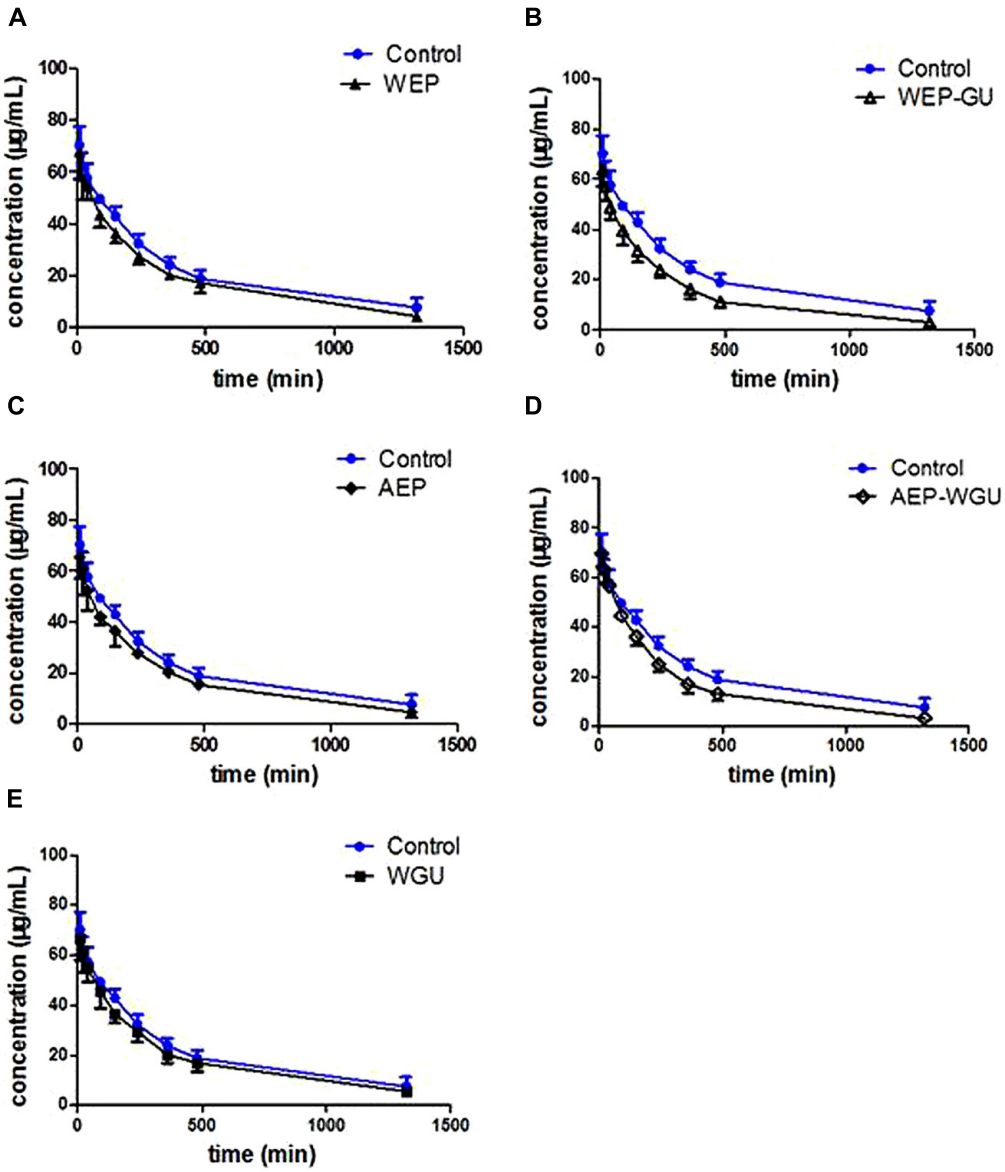
**Mean plasma concentration–time curve of tolbutamide after treatment. (A)** Control and WEP; **(B)** Control and WEP-GU; **(C)** Control and AEP; **(D)** Control and AEP-WGU; **(E)** Control and WGU; (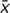
_±_ s, *n* = 6).

Pharmacokinetic profiles of tolbutamide after different treatment were used to describe the activity of CYP2C9. Mean plasma concentration time curves of tolbutamide in different groups are presented in **Figure 1**, which revealed that the clearance of tolbutamide in other treatment group were faster than Control. The effects of different treatment on pharmacokinetic parameters of tolbutamide in rats are presented in **Table 1**.

**Table 1:** Main pharmacokinetic parameters of tolbutamide in rats (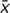
_±_ s, *n* = 6).

Parameters	Control	WGU	WEP	AEP	WEP-GU	AEP-WGU
AUC_0-1320__min_/μg min mL^-1^	16871.8 ± 1425.6	15614.6 ± 1823.8	15212.0 ± 1686.5	15023.3 ± 1092.1^∗^	13172.8 ± 1768.8^∗∗#^	14603.0 ± 1274.4^∗Δ^
AUC_0-∞_/μg min mL^-1^	24602.7 ± 3360.0	21899.7 ± 3919.3	19740.3 ± 2421.8^∗^	20867.1 ± 1139.6^∗^	16513.1 ± 2679.7^∗∗∗^	17910.5 ± 2896.9^∗∗Δ^
t_1/2_/min	275.6 ± 66.1	271.2 ± 44.2	231.1 ± 28.4	268.7 ± 26.0	212.4 ± 17.8^∗Δ^	196.7 ± 43.2^∗♢Δ^
MRT_0-1320__min_/min	179.0 ± 17.8	181.1 ± 7.9	180.1 ± 5.3	181.7 ± 7.5	170.7 ± 5.9^#Δ^	167.9 ± 9.6^♢Δ^
MRT_0-∞_/min	404.3 ± 81.3	394.6 ± 60.0	372.0 ± 48.7	380.0 ± 42.0	299.0 ± 23.8^∗##Δ^	297.6 ± 52.6^∗♢ΔΔ^
Vz/L kg^-1^	0.32 ± 0.04	0.36 ± 0.26	0.34 ± 0.04	0.37 ± 0.04	0.38 ± 0.04^Δ^	0.32 ± 0.02^♢^
CLz/L min^-1^ kg^-1^	0.001	0.001	0.001	0.001	0.001	0.001

*^∗^*P*< 0.05, ^∗∗^*P*< 0.01, ^∗∗∗^*P*< 0.001 compared with Control group.*

*^#^*P*< 0.05, ^##^*P*< 0.01 compared with WEP group.*

*^♢^*P*< 0.05 compared with AEP group.*

*^Δ^*P*< 0.05, ^ΔΔ^*P*< 0.01 compared with WGU group.*

Compared with Control, the pharmacokinetic parameters (AUC_0-1320__min_, AUC_0-∞_, MRT_0-1320__min_, MRT_0-∞_ and *t*_1/2_) of tolbutamide showed no significant change in WGU, which showed that GU had no inductive or inhibitory effect on the activities of CYP2C9 after multiple oral administrations in rats. Compared with Control, the AUC_0-∞_ of WEP and AEP significantly decreased (*P* < 0.05), AUC_0-1320__min_ of AEP significantly decreased (*P* < 0.05). The result showed that CYP2C9 activity was significantly induced by single use of EP. MRT_0-∞_ and *t*_1/2_ of WEP-GU decreased and had a very significant difference (*P* < 0.01) compared with Control, AUC_0-∞_ decreased and had a extremely significant difference (*P* < 0.001) compared with Control. It revealed that the co-decoction of EP and GU could induce the activity of CYP2C9. The *t*_1/2_ and MRT_0-∞_ of AEP-WGU all decreased significantly (*P* < 0.05), AUC_0-1320__min_ decreased very significantly (*P* < 0.01), AUC_0-∞_ decreased extremely significantly (*P* < 0.001) compared with Control, which showed that the co-administration of AEP and WGU could also induce the activity of CYP2C9. Therefore, WGU had no significant effect on the activity of CYP2C9. Both WEP and AEP could induce the activity of CYP2C9, and both the co-decoction of EP and GU and co-administration of AEP and WGU could also induce the activity of CYP2C9.

Compared with WEP, AUC_0-1320__min_ and MRT_0-1320__min_ of WEP-GU decreased very significantly (*P* < 0.01) and MRT_0-∞_ decreased extremely significantly (*P* < 0.001). Compared with WGU, *t*_1/2_, MRT_0-1320__min_ and MRT_0-∞_ of WEP-GU decreased significantly (*P* < 0.05). It showed that the co-decoction of EP and GU had a more potent inductive effect on the activity of CYP2C9 than single decoction solution of EP or GU. Compared with AEP, *t*_1/2_, MRT_0-1320__min_ and MRT_0-∞_ of AEP-WGU decreased significantly (*P* < 0.05). Compared with WGU, AUC_0-1320__min_, AUC_0-∞_, MRT_0-1320__min_, and *t*_1/2_ of AEP-WGU decreased significantly and MRT_0-∞_ decreased very significantly (*P* < 0.01). It showed that the co-administration of AEP and WGU had a more potent inductive effect on the activity of CYP2C9 than single administration of AEP or WGU.

**FIGURE 2:**
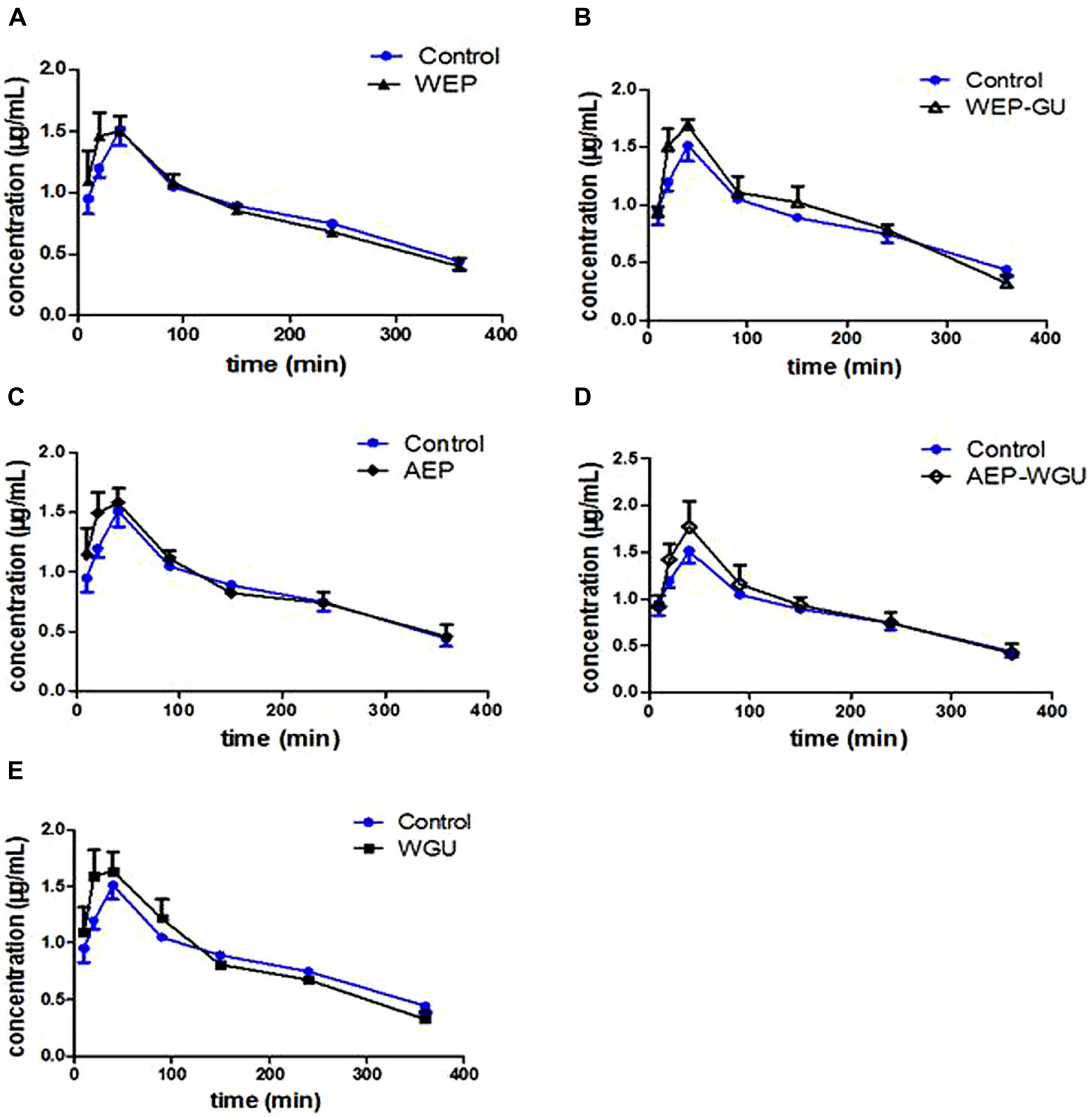
**Mean plasma concentration–time curve of 4-hydroxytolbutamide after treatment. (A)** Control and WEP; **(B)** Control and WEP-GU; **(C)** Control and AEP; **(D)** Control and AEP-WGU; **(E)** Control and WGU; (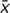
_±_ s, *n* = 6).

Mean plasma concentration time curves of 4-hydroxytolbuta- mide in different groups are presented in **Figure 2**. The effects of different treatment on pharmacokinetic parameters of 4-hydroxytolbutamide in rats are presented in **Table 2**.

**Table 2:** Main pharmacokinetic parameters of 4-hydroxy tolbutamide in rats (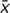
_±_ s,* n*= 6).

Parameters	Control	WGU	WEP	AEP	WEP-GU	AEP-WGU
AUC_0-1320__min_/μg min mL^-1^	488.3 ± 91.1	602.4 ± 69.5	634.1 ± 27.8^∗^	580.3 ± 135.2	628.5 ± 35.1^∗^	683.1 ± 167.7^∗^
AUC_0-∞_/μg min mL^-1^	506.9 ± 85.7	667.0 ± 125.7^∗^	687.5 ± 75.0^∗^	651.9 ± 168.9	715.9 ± 113.4^∗∗^	811.0 ± 199.9^∗∗^
t_1/2_/min	243.3 ± 62.7	372.0 ± 159.3	343.6 ± 150.4	373.2 ± 161.1	390.6 ± 215.2	524.8 ± 339.3
MRT_0-1320__min_/min	340.7 ± 133.0	367.0 ± 42.9	439.2 ± 76.9	340.8 ± 93.2	387.7 ± 41.6	421.8 ± 59.1
MRT_0-∞_/min	476.3 ± 260.2	527.4 ± 141.5	654.7 ± 152.4	490.1 ± 185.1	599.1 ± 171.3	770.2 ± 324.3
Vz/L kg^-1^	6.9 ± 1.5	7.75 ± 2.14	7.04 ± 2.43	7.86 ± 1.98	7.45 ± 3.35	9.2 ± 5.3
CLz/L min^-1^ kg^-1^	0.020 ± 0.004	0.016 ± 0.003	0.015 ± 0.001	0.017 ± 0.006	0.013 ± 0.004^∗^	0.013 ± 0.003

*^∗^*P*< 0.05, ^∗∗^*P*< 0.01 compared with Control group.*

Compared with Control, the AUC_0-∞_ of WGU increased significantly (*P* < 0.05); AUC_0-1320__min_ and AUC_0-∞_ of WEP increased significantly (*P* < 0.05); and AUC_0-1320__min_ of WEP-GU and AEP-WGU increased significantly (*P* < 0.05), which revealed that all the treatment could induce the activity of CYP2C9 to some extent.

### RESULT OF *IN VITRO* EXPERIMENT

**FIGURE 3:**
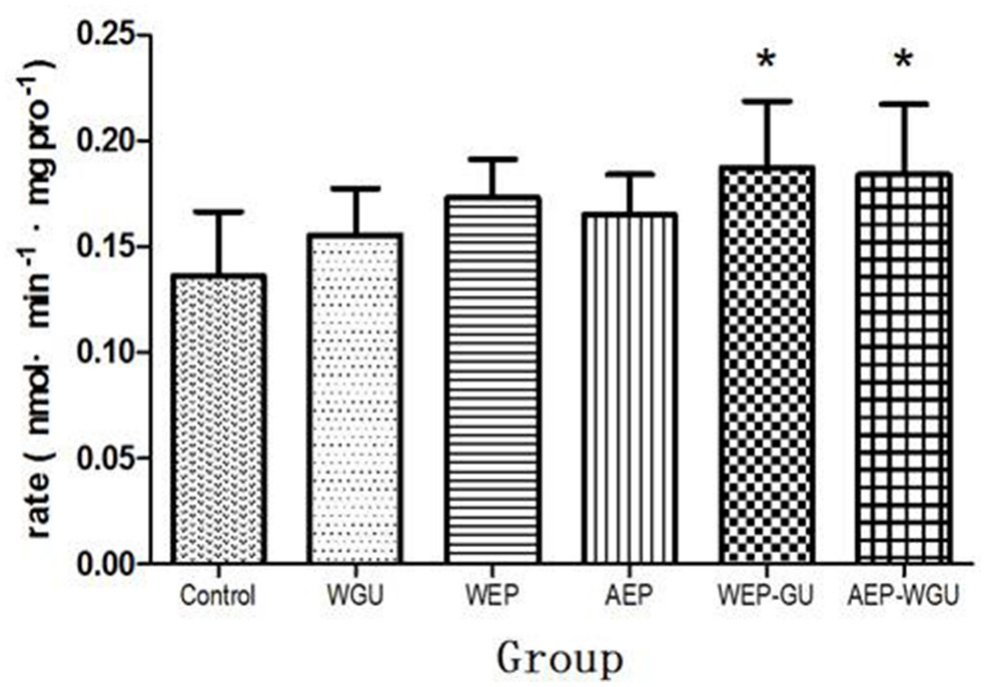
**Activities of CYP 2C9 in liver microsomes (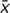
_±_ s, *n* = 6).** ^∗^*P* < 0.05 compared with control.

After microsomal incubation, the formation of 4-hydroxytolbut- amide were determined and thus the formation rate of 4-hydroxytolbutamide of different treatment group were calculated and used as the indicator of the activity of CYP2C9 *in vitro*, as was presented in **Figure 3**. Compared to Control (0.137 ± 0.030), the formation rate (nmol/min mgpro) of WGU (0.156 ± 0.022), WEP (0.173 ± 0.018), and AEP (0.165 ± 0.019) had the tendency of promotion, but there was no statistically significant difference. WEP-GU (0.187 ± 0.031) and AEP-WGU (0.184 ± 0.033) increased significantly (*P* < 0.05), which revealed the corresponding treatment could induce the activity of CYP2C9.

## DISCUSSION

CYP2C9 is an enzyme of the CYP450 superfamily of monooxygenases (Johnson et al., 2005). Some other isoforms of CYP enzymes, such as CYP3A4, CYP2D6, and CYP2C19, play important roles in drug metabolism (Zanger and Schwab, 2013) as well, but accumulating evidence indicates that CYP2C9 ranks amongst the most important drug metabolizing enzymes in human (Miners and Birkett, 1998), so we chose the single isoform of metabolic enzymes (CYP2C9) to study the herb–herb interaction of Glycyrrhizae Radix and Euphorbiae Pekinensis Radix. Substrates for CYP2C9 include fluoxetine, losartan, phenytoin, tolbutamide, torsemide, S-warfarin, and numerous non-steroidal anti-inflammatory drugs (NSAIDs) (Leemann et al., 1993; Takahashi and Echizen, 2001; Zhou et al., 2010). In human tolbutamide is metabolized almost exclusively along a single pathway. Methylhydroxylation to form 4-hydroxytolbutamide is the initial and rate-limiting step; subsequent oxidation of dehydrogenases produces carboxytolbutamide (Nelson and O’Reilly, 1961; Thomas and Ikeda, 1966). Overall, this pathway accounts for up to 85% of tolbutamide clearance in human (Thomas and Ikeda, 1966; Veronese et al., 1990). There is overwhelming evidence that CYP2C9 is solely responsible for the hydroxylation of tolbutamide. Some isoforms of CYP2C have high relevance with CYP2C9, according to the literature (Lasker et al., 1998), both CYP2C9 and CYP2C19 were efficient tolbutamide hydroxylases, but the microsomal CYP2C19 levels were substantially less than CYP2C9 levels which suggested that the former P450 played a nominal role in hepatic tolbutamide metabolism, thus tolbutamide was widely accepted as a probe substrate for assessment of hepatic CYP2C9 activity, both *in vitro* and *in vivo*.

In this study, the classics probe substrate tolbutamide was used to evaluate the effect of single Euphorbiae Pekinensis Radix, single Glycyrrhizae Radix, and the combination of both on the activity of CYP2C9. According to TCM (Commission, 2010), Glycyrrhizae Radix is used in form of water decoction and Euphorbiae Pekinensis Radix is used in form of water decoction, pill, and powder. Thus water extract of Glycyrrhizae Radix, both water extract and alcohol extract of Euphorbiae Pekinensis Radix were prepared for this research. The combination of both herbs was prepared by (1) water extract of the mixed Euphorbiae Pekinensis Radix and Glycyrrhizae Radix (1:1, co-decoction); (2) mixture of water extract of Glycyrrhizae Radix and alcohol extract of Euphorbiae Pekinensis Radix (1:1, co-administrated). *In vivo* study demonstrated that Glycyrrhizae Radix (water extract), Euphorbiae Pekinensis Radix (both water extract and alcohol extract), and combination of two herbs (both co-decoction and co-administrated) could induce the activity of CYP2C9. Compared to single herb, combination of Glycyrrhizae Radix and Euphorbiae Pekinensis Radix had a more potent induction effect on the activity of CYP2C9. *In vitro* study revealed that single herb showed no statistically significant effects on the activity of CYP2C9, while the combination of both herbs could induce the activity of CYP2C9. In general, both *in vivo* and *in vitro* study indicated that the combination of Glycyrrhizae Radix and Euphorbiae Pekinensis Radix could induce the activity of CYP2C9 to a high level.

According to drug–drug interaction based on CYP450, inhibition of CYPs can lead to increased plasma levels of drugs that are substrates for CYPs and thus can cause toxicity. In contrast, induction of this enzyme can result in decreased plasma levels of these drugs and consequently reduce their efficacy (Guo et al., 2014). As CYP2C9 was an important drug-metabolizing enzyme, the induction effect of combination of Glycyrrhizae Radix and Euphorbiae Pekinensis Radix on activity of CYP2C9 may speed up the metabolism of major active ingredients in Glycyrrhizae Radix and Euphorbiae Pekinensis Radix, as well as other herbs in the prescriptions, thus lower the plasma concentration of the these active ingredients and also the concentration of the ingredients at the target site, resulting in a poor efficacy. To further elucidate that, the enzymes involved in the metabolism of the active ingredients in Glycyrrhizae Radix and Euphorbiae Pekinensis Radix needs to be studied.

Moreover, the involvement of CYP2C9 has been shown in the activation of several carcinogens, such as polycyclic aromatic hydrocarbons and heterocyclic aromatic amines (García-Martín et al., 2002). Beside the well-known role in metabolizing and activating of pro-carcinogens, CYP2C9 seems to be relevant for early esophageal cancer development by promoting tumor cell proliferation (Schmelzle et al., 2011) and altered CYP2C9 metabolism may play a relevant role in lung carcinogenesis (García-Martín et al., 2002). The induction of CYP2C9 activity by combination of Glycyrrhizae Radix and Euphorbiae Pekinensis Radix may lead to a high risk for cancer.

## CONCLUSION

It was concluded that the combination of Glycyrrhizae Radix and Euphorbiae Pekinensis Radix were capable of significantly inducing CYP2C9 metabolic activity *in vitro* and *in vivo*. If the major active ingredients in Glycyrrhizae Radix and Euphorbiae Pekinensis Radix are mainly metabolized by CYP2C9, it will result in a poor efficacy. Moreover, these two herbs therefore, might have the potential to interact with other conventional medicines metabolized and eliminated from the body by CYP2C9. Although we are aware that our results are somewhat preliminary, they indicate at least a mechanism of herb–herb interaction of Euphorbiae Pekinensis Radix and Glycyrrhizae Radix.

## Conflict of Interest Statement

The reviewer Dr. Kevin Yue Zhu declares that, despite being affiliated to the same institution as the authors, the review process was handled objectively and no conflict of interest exists. The authors declare that the research was conducted in the absence of any commercial or financial relationships that could be construed as a potential conflict of interest.
